# Therapeutic Potential of a Novel Lytic Phage, vB_EclM_ECLFM1, against Carbapenem-Resistant *Enterobacter cloacae*

**DOI:** 10.3390/ijms25020854

**Published:** 2024-01-10

**Authors:** Saieeda Fabia Ali, Soon-Hian Teh, Hsueh-Hui Yang, Yun-Chan Tsai, Huei-Jen Chao, Si-Shiuan Peng, Shu-Chen Chen, Ling-Chun Lin, Nien-Tsung Lin

**Affiliations:** 1 Master Program in Biomedical Sciences, School of Medicine, Tzu Chi University, No. 701, Sec. 3, Zhongyang Rd., Hualien 97004, Taiwan; 109329106@gms.tcu.edu.tw; 2 Division of Infectious Diseases, Department of Internal Medicine, Hualien Tzu Chi Hospital, Buddhist Tzu Chi Medical Foundation, No. 707, Sec. 3, Zhongyang Rd., Hualien 97002, Taiwan; jimmyteh@tzuchi.com.tw; 3 Department of Medical Research, Hualien Tzu Chi Hospital, Buddhist Tzu Chi Medical Foundation, No. 707, Sec. 3, Zhongyang Rd., Hualien 97002, Taiwan; hhyang@tzuchi.com.tw; 4 Department of Life Sciences, College of Medicine, Tzu Chi University, No. 701, Sec. 3, Zhongyang Rd., Hualien 97004, Taiwan; 108711108@gms.tcu.edu.tw; 5 Department of Laboratory Medicine, Hualien Tzu Chi Hospital, Buddhist Tzu Chi Medical Foundation, No. 707, Sec. 3, Zhongyang Rd., Hualien 97002, Taiwan; jhj03070@gmail.com (H.-J.C.); pengcity@yahoo.com.tw (S.-S.P.); k751019@gmail.com (S.-C.C.)

**Keywords:** *Enterobacter cloacae*, multidrug resistance, bacteriophage, phage therapy, *Karamvirus*, antimicrobial resistance

## Abstract

The global rise of multidrug-resistant *Enterobacter cloacae* strains, especially those that are resistant to carbapenems and produce metallo-β-lactamases, poses a critical challenge in clinical settings owing to limited treatment options. While bacteriophages show promise in treating these infections, their use is hindered by scarce resources and insufficient genomic data. In this study, we isolated ECLFM1, a novel *E. cloacae* phage, from sewage water using a carbapenem-resistant clinical strain as the host. ECLFM1 exhibited rapid adsorption and a 15-min latent period, with a burst size of approximately 75 PFU/infected cell. Its genome, spanning 172,036 bp, was characterized and identified as a member of *Karamvirus*. In therapeutic applications, owing to a high multiplicity of infection, ECLFM1 showed increased survival in zebrafish infected with *E. cloacae*. This study highlights ECLFM1’s potential as a candidate for controlling clinical *E. cloacae* infections, which would help address challenges in treating multidrug-resistant strains and contribute to the development of alternative treatments.

## 1. Introduction

The emergence of multidrug-resistant (MDR) clinical isolates represents a significant global issue, having critical clinical implications in community and hospital settings [1]. MDR strain infections contribute to increased mortality rates, prolonged hospital stays, and escalated healthcare expenses, posing a significant burden on global public health. This issue is particularly important in the current COVID-19 era, in which excessive antibiotic usage has contributed to a significant rise in MDR cases [2].

The *Enterobacter cloacae* complex (ECC) is a group of gram-negative bacteria including species such as *E. cloacae*, *E. hormaechei*, *E. asburiae*, *E. kobei*, *E. ludwigii*, *E. nimipressuralis*, and *E. mori*, which belong to the *Enterobacteriaceae* family [3]. These bacteria are cosmopolitan, facultative anaerobic, and appear as rod-shaped cells with rounded ends when observed under a microscope [4]. ECC is the third most prevalent *Enterobacteriaceae* species, following *Klebsiella pneumoniae* and *Escherichia coli*, and is associated with both nosocomial and community-acquired infections, including pneumonia, urinary tract infections, intra-abdominal infections, and bacteremia [5]. The ECC is recognized as the second most prevalent genus with resistance to β-lactam antibiotics in the *Enterobacteriaceae* family, and exhibits inherent resistance to first- and second-generation cephalosporins, primarily attributed to the overexpression of efflux pumps and chromosomally-encoded class C β-lactamases [6]. These mechanisms enable the ECC to withstand antibiotic activity and contribute to their overall resistance profile [7]. Further, they are versatile carriers of extended-spectrum β-lactamase (ESBL) producing genes (such as *bla*_TEM_, *bla*_SHV_, and *bla*_CTX-M_) and carbapenemase (such as *bla*_OXA-48_, *bla*_VIM_, *bla*_IMP_, *bla*_KPC_, and *bla*_NDM-1_) via horizontal gene transfer [8]. Hence, MDR emergence, including resistance to the last-resort carbapenems meropenem, imipenem, and ertapenem, has been classified by the World Health Organization as a priority for antimicrobial research and development [9].

Over the past three decades, ECC has emerged as a challenging pathogen in various countries, including Spain, the USA, Japan, and China [10,11,12,13,14]. A major concern associated with ECC infections is the rising prevalence of carbapenem-resistant ECC (CR-ECC) because available treatment options are limited, causing difficulties in managing these infections. The emergence and spread of CR-ECC strains have further complicated the clinical management of ECC infections, highlighting the urgent need for alternative therapeutic strategies and infection control measures. 

In Taiwan, the prevalence of nosocomial infections stemming from ECC is steadily increasing and is accompanied by a concurrent surge in drug resistance. According to the Taiwan Surveillance Antimicrobial Resistance Trends Study (SMART) conducted from 2016 to 2018, ECC isolates exhibited a higher rate of non-susceptibility to the antibiotic ertapenem compared to isolates of *E. coli* or *K. pneumoniae* [15]. Particularly in intensive care unit patients, ECC is reported to have the highest resistance rate to carbapenems, increasing annually [16]. This escalating clinical challenge underscores the urgency of exploring alternative green approaches that are distinct from conventional antibiotics to combat antibiotic resistance and minimize ECC infections. The results obtained from various in vitro and in vivo studies highlight the potential of utilizing phage therapy to address bacterial infections [17]. These findings have encouraged ongoing investigations on the utility of phage therapy as a potential cure for bacterial infections [18]. 

Bacteriophage/phage is a virus that infects and replicates only in bacteria and is species/strain-specific, recognizing their hosts alone to infect and eliminate the host bacteria [19]. Therefore, phage characterization for therapeutic applications, which involves isolating potentially lytic phages, observing morphology, studying replication strategies (lytic or lysogenic), understanding lytic host efficiency, determining presence of virulence or resistance genes, and analyzing bactericidal/bacteriostatic efficiency of the in vivo system, is necessary [20]. Despite the global acknowledgment of and attention directed towards phage therapy, the co-evolutionary dynamics between phages and their host bacteria emphasize the need to isolate and comprehensively study phages that specifically target regionally prevalent drug-resistant strains. 

In contrast to research conducted on other members of the *Enterobacteriaceae* family, such as *E. coli* and *K. pneumoniae*, there has been relatively less research on exploring phages and prospective clinical applications targeting the ECC. This is especially true in Taiwan, where limited phage resources and insufficient genomic data pose obstacles to the treatment of ECC. Therefore, further research and exploration of phages specifically targeting ECC strains is required to form a basis for future development of effective treatments against these dangerous infections. The isolation and description of ECC phages is crucial in understanding their role in ecological functioning, interactions, and co-evolution with the host. In this study, we characterized a lytic phage isolated from a sewage sample collected in Hualien, Taiwan, specifically targeted towards *E. cloacae*, the most commonly identified species of ECC in clinical infections [21]. This phage was also tested for the treatment efficacy of *E. cloacae*-infected zebrafish.

## 2. Results

### 2.1. Morphological Characterization of Phage vB_EclM_ECLFM1

**Figure 1:**
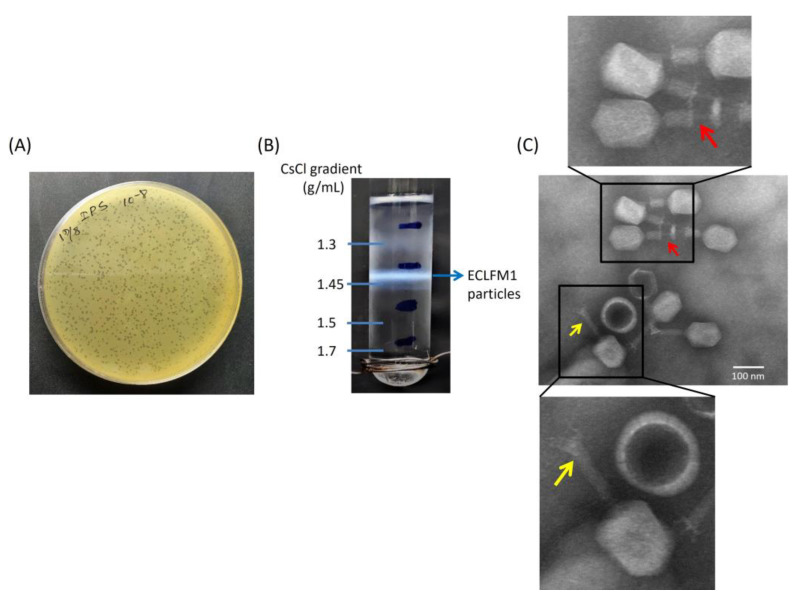
Morphology and buoyant density of ECLFM1. (**A**) Plaque morphology on a plate containing bacterial overlay with scale bar of 1 cm. (**B**) CsCl gradient revealed that the buoyant density of ECLFM1 is 1.45 g/mL. (**C**) Electron micrograph of ECLFM1 virions with scale bar of 100 nm. The CsCl-purified viral particles were negatively stained with 2% uranyl acetate and visualized at 120K-fold magnification. The phage particle has a constricted tail sheath (yellow arrow) and a tail tube protruding from the tip of the tail (red arrow), which can be seen as a myovirus.

To develop phage therapy for infections caused by carbapenem-resistant clinical strains of *E. cloacae*, we opted for clinical strain ECL73134 as the host. This particular strain exhibits resistance to kanamycin and tetracycline and demonstrates intermediate resistance to ciprofloxacin. Notably, it carries the *bla*_IMP-8_ gene, which encodes a metallo-β-lactamase, rendering it resistant to meropenem, ertapenem, and imipenem. The ECLFM1 phage was isolated from sewer wastewater samples around Tzu Chi Hospital in Hualien, Taiwan by spot tests using ECL73134 as the bacterial lawn. ECLFM1 produced small, morphologically similar clear plaques on double-layer agar plates with no surrounding halo (Figure 1A). For characterization, ECLFM1 was enriched via repeated plaque purification, and a stock of 10^11^ PFU/mL was prepared and subsequently subjected to ultracentrifugation in a discontinuous cesium chloride (CsCl) gradient (ρ = 1.7, 1.5, 1.45, and 1.3 g/mL), and phage particles were found to band in the 1.45 g/cm^3^ block (Figure 1B).

Transmission electron microscopy (TEM) observation of phage morphology showed that ECLFM1 was a myovirus belonging to class Caudoviricetes with a head-to-tail geometry, an icosahedral head of approximately 118 nm, and a contractile tail of 118 nm in length; the neck, base plate, spikes, or fibers can be clearly seen on the phage particle (Figure 1C).

### 2.2. Host-Range Activity Determination and Efficiency of Plating (EOP) Analysis

ECLFM1’s ability to lyse pathogenic *E. cloacae* strains was assayed via spot test using the lawns of 123 different clinical *E. cloacae* strains isolated from hospital samples. After 16 h, 96 (78%) tested strains showed a spotted inhibition halo, indicating high phage infectivity towards *E. cloacae* clinical isolates; however, bacteria other than *E. cloacae*, such as *K. pneumoniae*, *P. aeruginosa*, *E. coli*, and *A. baumannii*, were not susceptible to ECLFM1, indicating phage specificity to the host.

**Figure 2:**
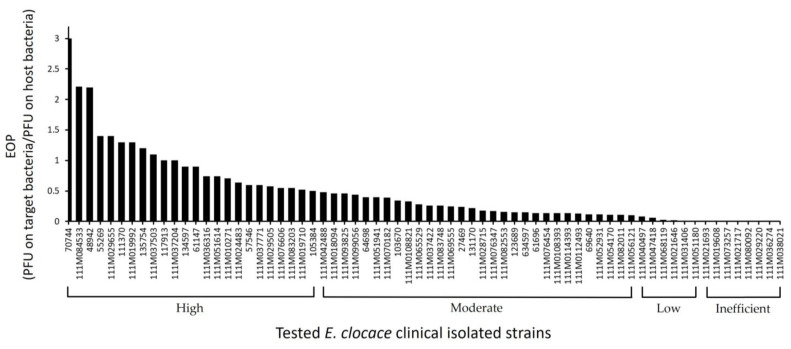
Efficacy evaluation of ECLFM1 against 68 susceptible clinical isolates of *E. cloacae*. The efficiency of plating (EOP) was determined by comparing the number of plaques generated in the host strain ECL73134. The vertical lines represent EOP values; the markers on the horizontal line correspond to individual clinical strains of *E. cloacae*.

EOP analysis was performed on ECLFM1-susceptible isolates from the spot test. Although spot test results revealed clear plaques on the bacterial lawn of all susceptible *E. cloacae* isolates, EOP results showed variation in phage lysis efficiency. In total, 24, 30, and 6 strains exhibited high (EOP ≥ 0.5), moderate (0.1 ≤ EOP < 0.5), and low (0.001 < EOP < 0.1) productive EOP values, respectively, whereas eight *E. cloacae* isolates were inefficient (EOP ≤ 0.001) against ECLFM1 infection (Figure 2). Some strains did not exhibit plaques on the bacterial lawns.

### 2.3. Biological Characterization of ECLFM1

**Figure 3:**
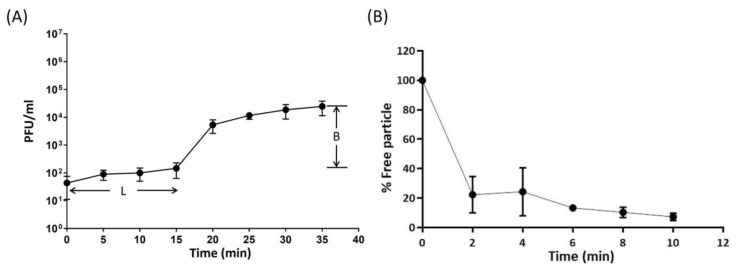
One-step growth and adsorption curve of ECLFM1. (**A**) One-step growth of ECLFM1 on ECL73134. The latent period of ECLFM1 was approximately 15 min, and the burst size was 75 PFU/infected cells. L: latent period; B: burst size. (**B**) Adsorption of ECLFM1 to ECL73134. Approximately 80% of ECLFM1 particles were adsorbed onto the cells after 2 min, and nearly 90% were absorbed after 10 min. These experiments were performed in triplicate and the data is shown as mean ± SEM.

The multiplication capacity, latency period, and burst size of ECLFM1 was determined via one-step growth experiment. The results indicated that ECLFM1 had a short latent period of 15 min, followed by a rise period of 20 min. A growth plateau was reached within 35 min, and the average burst size was calculated to be approximately 75 phage particles per infected bacterial cell (Figure 3A).

The absorption rate of ECLFM1 onto ECL73134 was investigated and the results showed that approximately 80% of the phage particles were adsorbed within 2 min, and more than 90% were adsorbed after 10 min (Figure 3B).

### 2.4. Bacteriolytic Activity In Vitro

**Figure 4:**
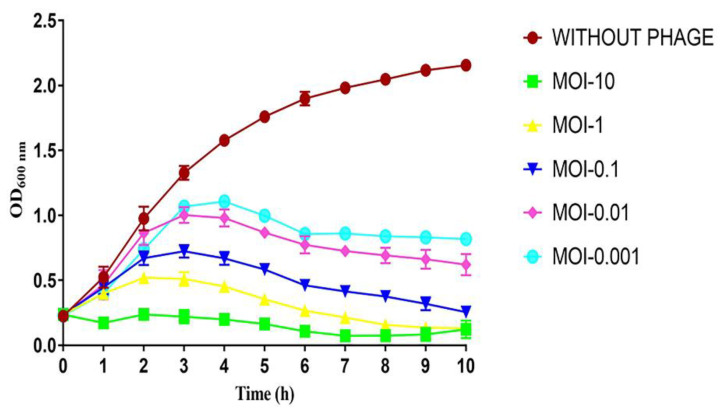
Bacteriolytic activity of ECLFM1 against ECL73134 at different MOIs. These experiments were performed in triplicate, and data are shown as mean ± SEM.

To evaluate the bacteriolytic activity of ECLFM1, different multiplicities of infection (MOIs) and corresponding bacterial growth were observed via monitoring the optical density at 600 nm (OD_600_). The results showed that the absorbance of the host without ECFLM1 infection continued to increase over 10 h, while the absorbance of the phage-infected culture gradually increased in an MOI-dependent manner during the first 3 h and gradually decreased between 3 and 10 h (Figure 4).

### 2.5. Determination of Phage Stability at Different pH, Temperatures, and Long-Term Storage

**Figure 5:**
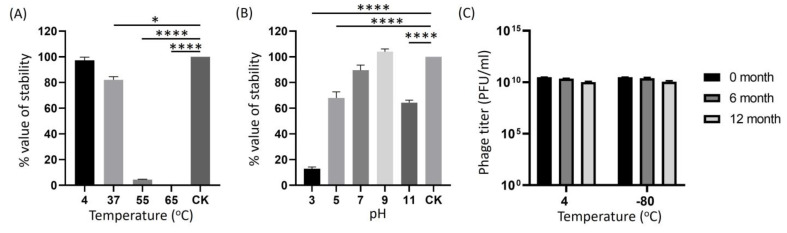
Biophysical stability of ECLFM1. (**A**) Thermal stability of ECLFM1. Phages were incubated for 1 h under different temperatures. (**B**) pH stability of ECLFM1. Phages were incubated for 1 h under different pH values. (**C**) Long-term storage stability of ECLFM1 at 4 °C and -80 °C for up to 12 months. These experiments were performed in triplicate, and data are shown as mean ± SEM. All experiments were based on the plaque-forming ability of the phage on the host lawn to assess infectious activity relative to the control under the aforementioned conditions. The results of panels (**A**,**B**) are expressed as the ratio of the tested phage titer to the original loading titer (CK). Significance was determined by one-way ANOVA with Dunnett’s multiple comparisons post-hoc test. Bars represent standard deviation (SD). Asterisks indicate significant differences (* *p* ≤ 0.05; **** *p* ≤ 0.0001).

To prepare ECLFM1 into a usable phage preparation, its infection stability was assessed by exposure to various external factors, including pH, temperature, and long-term storage. After 1 h of incubation at 4 °C, the ECFLM1 phage titer did not change significantly compared to the original loading concentration; it was slightly lower at 37 °C and significantly reduced to only 5% at 50 °C. However, the phage was completely inactivated at 65 °C, indicating that ECLFM1 is thermolabile (Figure 5A). ECLFM1 showed good stability from pH 7 to pH 9, whereas it lost significant lytic activity after incubation for 1 h below pH 5 and above pH 11 (Figure 5B). To test for long-term storage stability of the phage, crude lysates of ECLFM1 (ca. 3.0 × 10^10^ PFU/mL) were stored at 4 °C in SM buffer and −80 °C in SM buffer supplemented with 50% glycerol; phage titers were assayed at 6-month intervals. After 12 months, no significant loss in infectivity was observed in both conditions (Figure 5C). These findings suggest that both temperature conditions and storage solutions are effective in preserving the infectivity stability of ECLFM1 for a minimum of 1 year.

### 2.6. Genomic Analysis and Annotation

**Figure 6:**
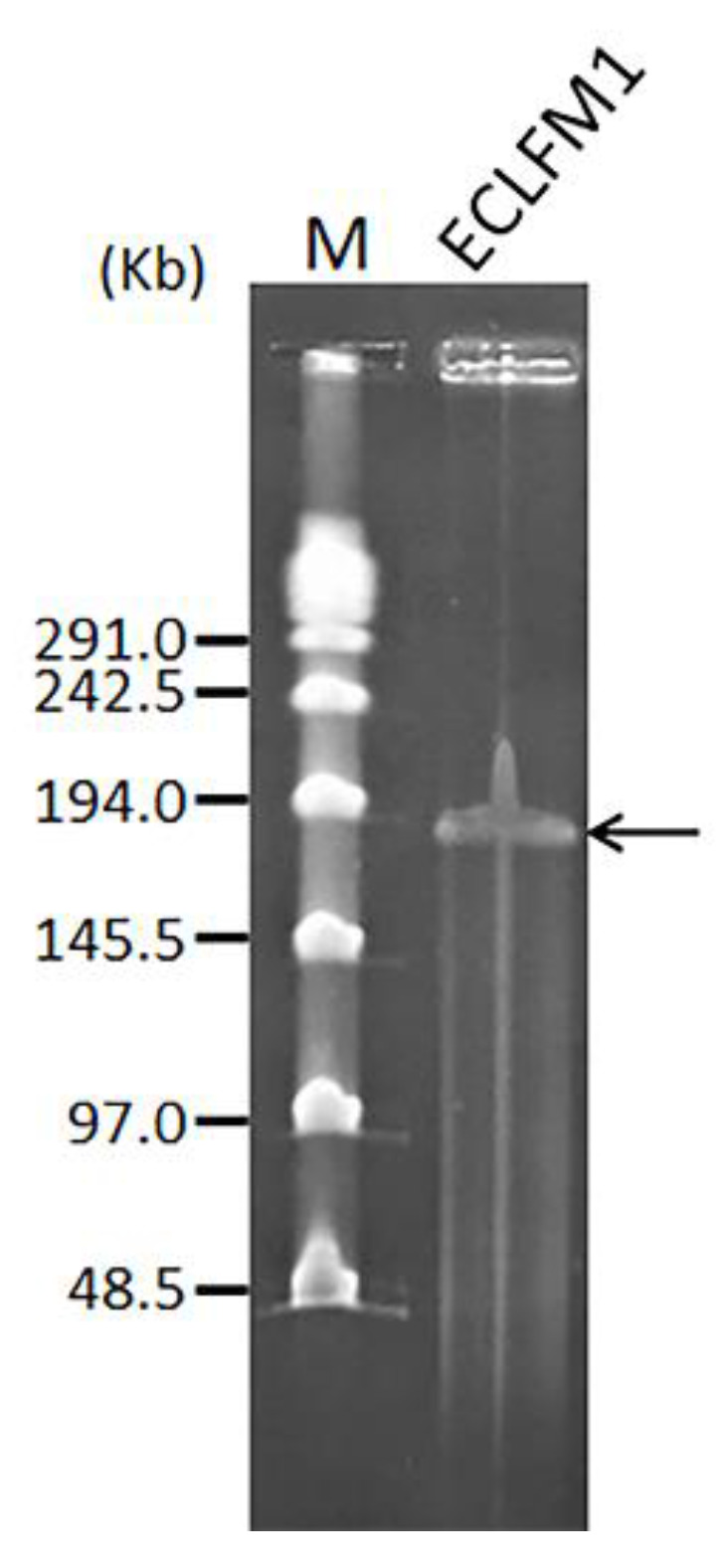
Estimated size of the ECLFM1 genome (black arrow) was determined by pulse field gel electrophoresis. Lane M is the λ PFG ladder (New England BioLabs, Ipswich, MA, USA).

The genome size of ECLFM1 was found to be 145.5–194 kb via pulse field gel electrophoresis (Figure 6). To further characterize the genome, we attempted to digest the phage genomic DNA using various enzymes, including HindIII, PstI, SspI, EcoRI, EcoRV, ScaI, and NdeI; however, none of these enzymes were able to digest the phage genomic DNA. The reason for this observation may be that the phage genome adapted according to the universal restriction modification system during the evolution process or that it carries a gene encoding a methyltransferase, which can modify the bases in one or more restriction sites; the phage may also contain unconventional bases as part of its genetic makeup.

**Figure 7:**
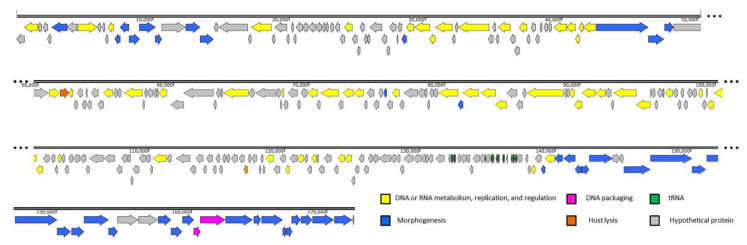
ECLFM1 genome organization. The coding DNA sequences (CDS) are represented as arrows, with colors differentiating the functional modules or regions. Positions of tRNA genes are marked with green arrows. Yellow arrows represent CDS related to DNA or RNA metabolism, replication, and regulation. Blue, orange, and grey arrows indicate CDS associated with morphogenesis, involved in host lysis, and with unknown functions, respectively. Pink arrows represent CDS related to DNA packaging.

Whole genome sequencing revealed that ECLFM1 had a genome size of 172,036 bp and contained 126-bp direct repeats at both ends. Additionally, the genome contained 19 tRNA genes and a GC content of 39.7%, lower than that of E. cloacae ATCC 13047 (54.79%) [22]. After conducting a BLASTN analysis, we found that the ECLFM1 genome was closely related to Enterobacter phage PG7 (NC_023561), with 95% coverage and 97.4% similarity. Further analysis of the complete ECLFM1 genome using PhageLeads [23] revealed the absence of genes associated with temperate or lysogenic life cycles, and virulent- and antibiotic-related genes. This indicates that ECLFM1 has a strictly lytic life cycle and does not carry genes conferring lysogeny or antibiotic resistance. A total of 288 open reading frames (ORFs) were predicted using RAST. Both RAST and manual BLASTp results revealed that 108 ORFs (37.5%) had their functions predicted. The remaining 180 ORFs were categorized into four groups based on their putative functions: DNA or RNA metabolism, replication, and regulation; DNA packaging; host lysis; morphogenesis. Proteins with unidentified functions were categorized as hypothetical proteins (Figure 7). The complete list of all the proteins is available in Table S1.

#### 2.6.1. DNA or RNA Metabolism, Replication, and Regulation

The DNA metabolism and replication module of ECLFM1 involves the coordinated action of multiple enzymes. A total of 35 ORFs in ECLFM1 are associated with DNA metabolism, replication, and regulation. Many of these proteins exhibit similarities to counterparts found in *Enterobacter* phages. The longest ORF in size is ORF_144, measuring 2709 bp (104.3 kDa) and it shares similarities with the DNA polymerase from *Enterobacter* phage myPSH1140 [24]. ORF_118 encodes a DNA helicase that can separate the DNA double helix into individual strands via ATP hydrolysis, and it shares 99.77% identity with *Enterobacter* PG7. ORF_76 encodes a phage ribonuclease H that cleaves RNA strands of hybrids and shows 100% similarity to *Enterobacter* PG7. ORF_148 encodes the phage DNA polymerase clamp loader subunit, which is involved in DNA repair, recombination, and replication, and it exhibits 100% similarity to *Enterobacter* PG7. Other significant ORFs include DNA helicase (ORF_8), DNA ligase (ORF_24), single-stranded DNA-binding protein (ORF_72), phage DNA topoisomerase large subunit (ORF_106), replication factor C small subunit (ORF_149), and others.

#### 2.6.2. DNA Packaging and Assembly 

The major terminase components, ORF_277 and ORF_278, are crucial for the ECLFM1 packaging module. Terminase enzymes consist of a small subunit and a large subunit that together encapsulate or package the viral genome into the viral procapsid. The large terminase subunit cleaves the DNA at the initiation site, whereas the small terminase subunit is identifies viral DNA. Energy required for packaging initiation and termination is provided by the ATPase activity of the large terminase subunit [25]. Additionally, ECLFM1 employs other functional proteins, such as DNA end protector protein (ORF_260) and other proteins, to facilitate the packaging and assembly of its genome.

#### 2.6.3. Host Lysis

Holin, a small membrane protein, is crucial in determining the fate of phage-infected bacteria. It triggers the rupture of the bacterial cell membrane at a specific time, allowing a lysozyme to break down the cell wall [26]. In ECLFM1, the putative holin (ORF_82) and Phage lysozyme R (ORF_226) are responsible for lysing bacterial cell walls. Additionally, the lysis inhibition accessory protein (ORF_36), lysis inhibition regulator membrane protein (ORF_207), and three other proteins promote cell wall lysis and facilitate phage genome integration into the host bacteria.

#### 2.6.4. Morphogenesis

ECLFM1 phage structure and morphogenesis involve the participation of at least 37 proteins, which contribute to various aspects of phage development and assembly. Particularly, six ORFs are associated with phage head-related proteins, ORF_274 and ORF_275 encode the phage neck protein, three ORFs are related to capsid scaffold proteins, 13 ORFs are involved in phage baseplate functions, and seven ORFs are associated with tail-related proteins (Table S1). These proteins are essential for phage head formation and stability, assist in tail fiber attachment during viral assembly, initiate infection via recognizing host cell receptors, and facilitate phage genome binding and ejection into the host. Several specific proteins are also involved in phage head development. These include the phage head assembly chaperone protein (ORF_37), phage head decoration protein (ORF_126), and phage prohead assembly protein (ORF_284). Capsid and scaffold proteins are encoded by ORF_6 and ORF_137, which provide structural support and defense for the overall phage structure. The tail fiber proteins (ORF_79, ORF_81, and ORF_280) and proximal components (ORF_77 and ORF_278) are involved in identifying and binding to receptors on the bacterial surface. 

In addition to the proteins directly involved in morphogenesis, the ECLFM1 phage contains unclassified proteins, such as ribonucleotide reductase, glutaredoxin, and thymidylate synthase. These proteins may serve other functions within the phage lifecycle or contribute to phage adaptation and survival in its host environment.

The possible functionality of each ORF described above was based on homology prediction analysis performed through Protein BLAST rather than on experimental results.

### 2.7. Structural Protein Profile of the ECLFM1 Phage

**Figure 8:**
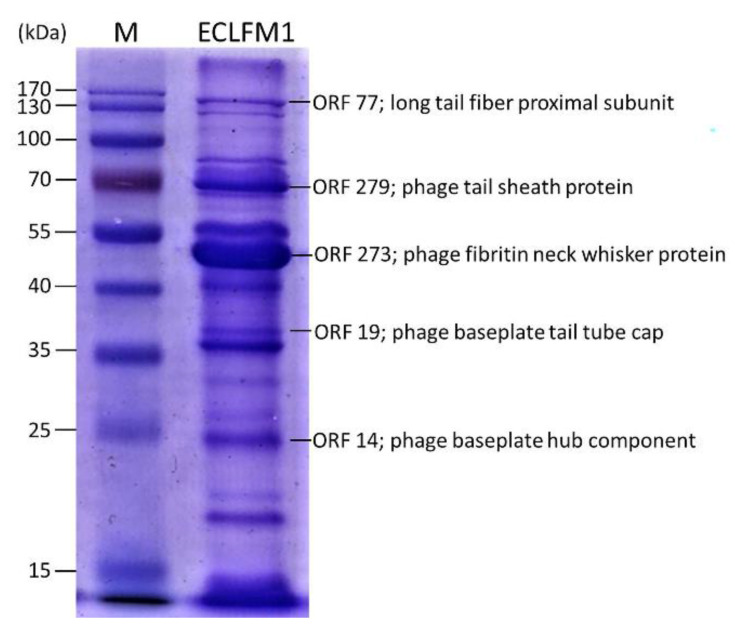
Sodium dodecyl sulphate-polyacrylamide gel electrophoresis (12%) of ECLFM1 virion proteins. Lanes: M, PageRulerTM Prestained Protein Ladder (Thermo Fisher Scientific, Waltham, MA, USA); ECLFM-1, ECLFM1 phage structural proteins. Relative migrations of molecular mass marker proteins are indicated on the left. Proteins identified via MS/MS are indicated on the right.

To further characterize ECLFM1, phage particle structural proteins were analyzed using sodium dodecyl sulfate (SDS)-polyacrylamide gel electrophoresis (12%). At least 16 protein bands were visualized in the gel (Figure 8), and the most abundant protein in ECLFM1 was an approximately 50-kDa virion component. Visible protein bands were recovered from the gel and further identified using liquid chromatography coupled to tandem mass spectrometry. The large protein was the long tail fiber proximal subunit (ORF_77), weighing 138.2 kDa. Additionally, the molecular masses of the phage tail sheath protein (ORF_279), phage baseplate tail tube cap (ORF_19), and phage baseplate hub component (ORF_14) were determined as 71 kDa, 38.2 kDa, and 24.3 kDa, respectively. The predominant protein was a phage fibritin neck whisker protein (ORF_273), which shared 98.09% of its similarity with *Cronobacter* phage Pet-CM3-4 [27]. Except for the above-mentioned specific bands that may correspond to structural proteins of other reported phages predicted by the genome sequence, the amino-acid sequences of the remaining bands were attributed to hypothetical proteins. These proteins have no homology to known phage structural proteins.

### 2.8. Comparative Genomics and Phylogenetic Analysis

**Figure 9:**
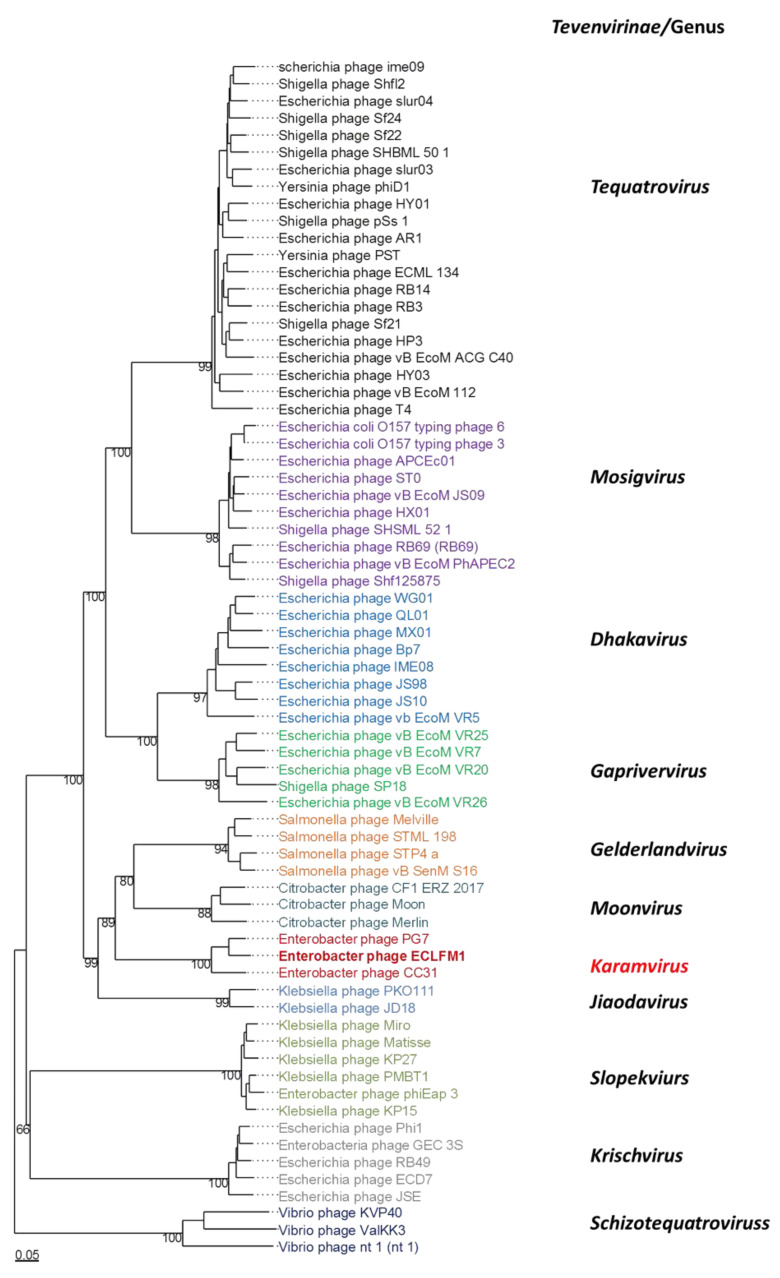
Phylogenetic analysis of whole genome of Enterobacter phage ECLFM1 and phages belonging to *Tevenvirinae* of *Straboviridae* using VICTOR. Different colors mark specific genera of *Tevenvirunae*.

**Figure 10:**
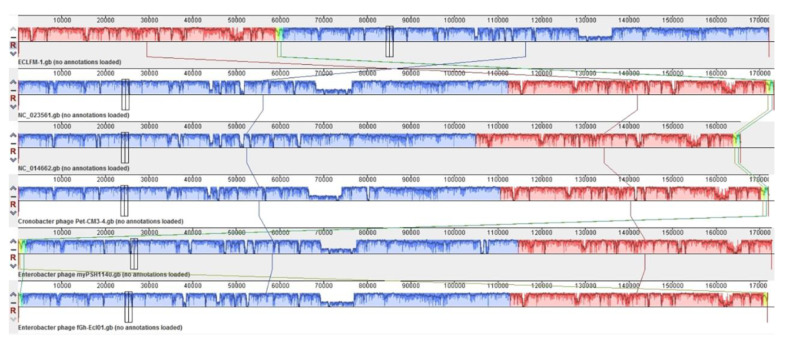
Multiple genome alignment of Enterobacter phage ECLFM1 and selective phages belonging to Karamvirus using Mauve software version 2.4.0 (http://asap.ahabs.wisc.edu/mauve/, accessed on 21 December 2014). The alignment results are displayed as a graphical representation, in which the bar height indicates sequence conservation levels in each genome region. Boxes with the same color represent local collinear blocks (LCB), which indicate homologous DNA regions shared by two or more chromosomes without sequence rearrangements.

**Figure 11:**
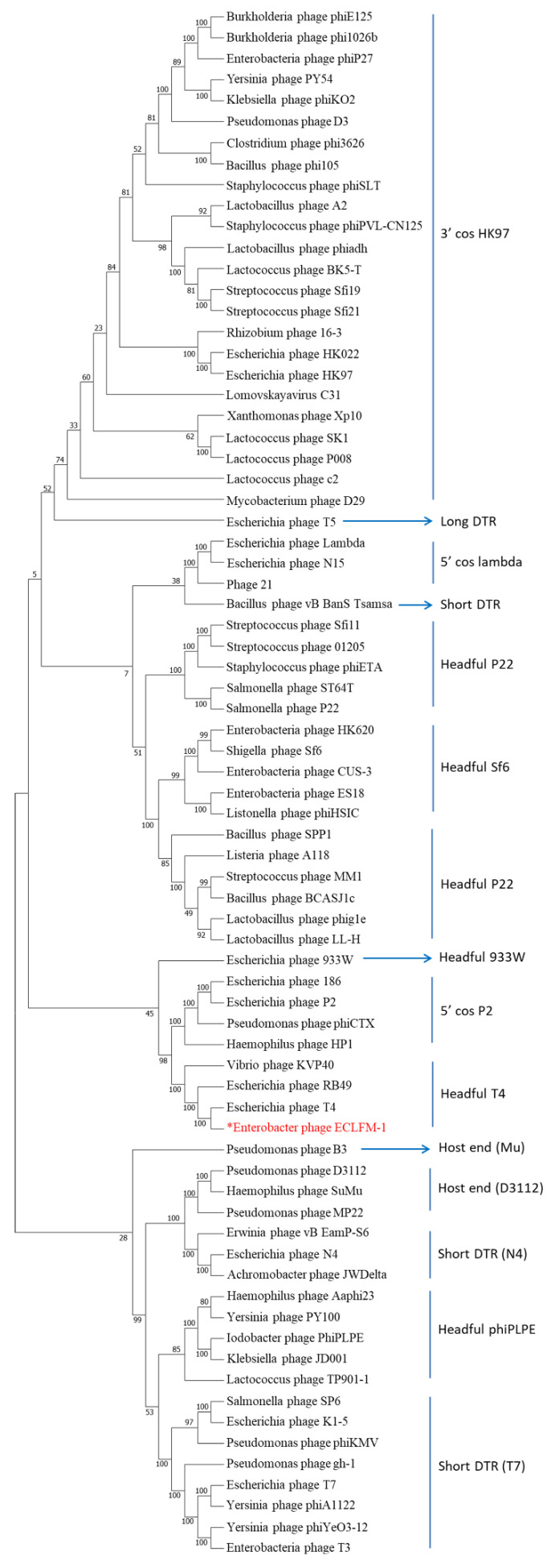
Phylogenetic tree of the large terminase subunit (ORF_278) encoded by the ECLFM1 phage. The amino acid sequences of the large terminase subunits were compared using the MEGA11 software version 11 and the tree was constructed using the neighbor-joining method with 1000 bootstrap replicates. The red words marked with an asterisk represent the relatedness of the ECLFM1 large terminase subunit to other phages in the phylogenetic tree.

Comparative analyses conducted using VIPTree (https://www.genome.jp/viptree/, accessed on 17 November 2023) demonstrated that ECLFM1 clustered with *Straboviridae* phages, and was more closely related to phages belonging to *Tevenvirinae*. VICTOR analysis (https://victor.dsmz.de, accessed on 1 November 2017) revealed that ECLFM1 shared the same clade with phages under the genus *Karamvirus* (Figure 9). Mauve alignment demonstrated that ECLFM1 had the same collinear genome arrangement with selective representatives of *Karamvirus*, including *Enterobacteria* phage CC3 (NC_014662.1), *Enterobacter* phage myPSH1140 (NC_055739.1), *Cronobacter* phage Pet-CM3-4 (NC_055726.1), *Enterobacter* phage PG7 (NC_023561.1), and *Enterobacter* phage fGh-Ecl0 (ON212265.1) (Figure 10). Moreover, the terminase large subunit (ORF_278) was analyzed to demonstrate how the virion DNA packaging system also shared the same headful T4 cluster (Figure 11). Thus, ECLFM1 was determined as a T4-like phage and placed under the genus *Karamvirus* in the subfamily *Tevenvirinae* and family *Straboviridae* as a novel member.

### 2.9. Successful Treatment of E. cloacae-Infected Zebrafish Using ECLFM1

**Figure 12:**
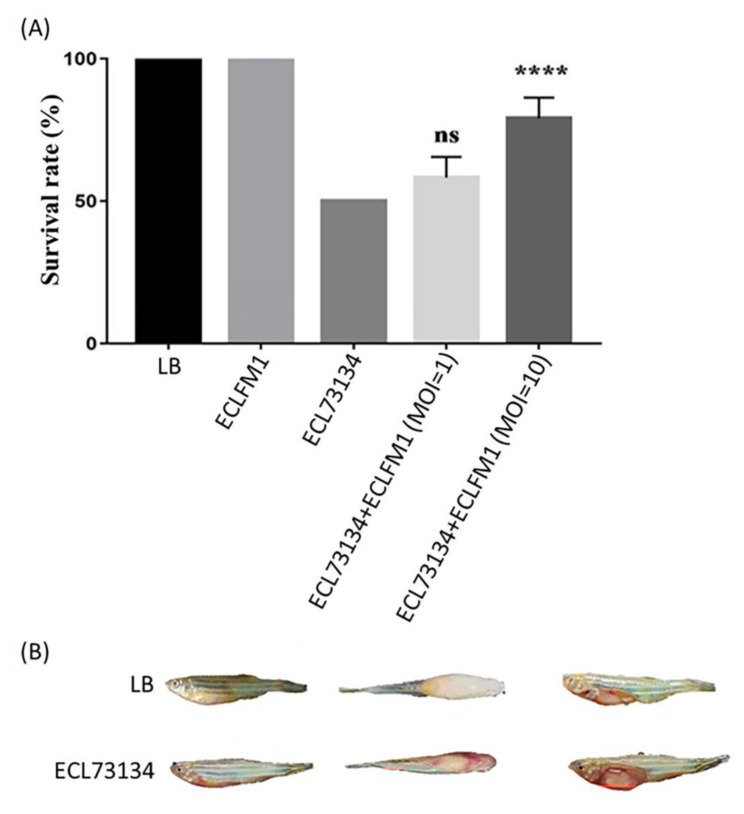
Pathogenicity of *E. cloacae* and therapeutic effect of ECLFM1 observed in the zebrafish model. (**A**) Fish survival was compared between the LD_50_ group and different multiplicities of infection (MOI). At MOI = 1, no significant difference in fish survival was observed compared to the LD_50_ group; however, at MOI = 10, a statistically significant difference was seen, indicating that treatment with a higher phage MOI increased fish survival rate. Each group consisted of eight zebrafish and the experiment was performed in triplicate, and data are shown as mean ± SEM. The significance of the differences between treated and was assessed by means of the log-rank and Gehan–Breslow–Wilcoxon tests using GraphPad Prism 9. “****” indicates *p* < 0.0001, and “ns” means no significance. (**B**) In zebrafish injected with Luria-Bertani broth medium, no symptoms were observed; however, zebrafish injected with the LD_50_ dose (3 × 10^8^ CFU/20µL) of ECL73134 exhibited swollen abdomen and superficial hemorrhage. The left, middle, and right panels show the side view, top view, and abdomen anatomy.

In vivo efficacy tests were performed for ECLFM1 using zebrafish as an animal model (Figure 12). The fish were infected with *E. cloacae* and treated with ECLFM1, followed by observation of survival for 24 h. During lethal dose 50% (LD_50_) assessment of *E. cloacae* in injected fishes, we observed that 50 (4/8), 38 (3/8), and 0% of fishes died after injection with a bacterial dose of 3 × 10^8^ CFU, 3 × 10^7^ CFU, and 3 × 10^6^ CFU, respectively. Based on these results, we determined the LD_50_ to be 3 × 10^8^ CFU. We then administered the LD_50_ dose of bacteria to the fish and subsequently treated them with phages. The fish survival rate was higher after phage administration in combination with ECL73134 compared to that after administration of the bacterium alone after 24 h. At MOI = 1 and MOI = 10, the fish survival rate was about 58% and 79%, respectively. In addition, we found that zebrafish infected with ECL73134 developed disease symptoms including abdominal swelling and superficial hemorrhage around the 24-h time point, whereas surviving fish injected with the medium (control group) and after phage treatment group did not show any disease symptoms at this time point.

## 3. Discussion

The ECC is a prominent nosocomial pathogen, particularly with the emergence of carbapenem-resistant strains. Many clinically relevant isolates of *E. cloacae* exhibit resistance to key β-lactam antibiotics, such as ampicillin and amoxicillin, posing significant challenges in their infection management, particularly in immunocompromised patients, and can result in serious health complications, including bacteremia, endocarditis, and mortality [28]. Specific lytic phages against clinical isolates of *E. cloacae* have been previously isolated [29,30,31,32,33] and assessed for their efficacy. ECC isolates in Taiwan showed greater resistance to ertapenem compared to *E. coli* and *K. pneumoniae* isolates [34]. This is a significant concern particularly in intensive care unit settings, wherein ECC has demonstrated the highest resistance rate to carbapenems among these bacterial pathogens. Furthermore, the ECC resistance rate to carbapenems has been increasing over time [35,36,37]. Hence, the investigation and development of alternative approaches to combat ECC infections is imperative. In this study, we characterized ECLFM1 as a novel T4-like lytic phage with a broad host range. A total of 96 (78%) tested strains responded to this phage, most of which were carbapenem-resistant *E. cloacae*. A broad host range is advantageous for therapeutic application because it allows for phage usage on many strains. Furthermore, the isolated phage did not contain antibiotic resistance genes and virulence factors, which may enhance the application availability. Belonging to the *Tevenvirinae* subfamily, which exhibits a strictly virulent life mode, ECLFM1 contains no integrases and lysogeny modules.

Bacterial cell lysis is the final stage in the phage reproductive cycle. Holin, a small membrane protein that triggers membrane rupture during a certain period, determines the destiny of phage-infected bacteria by allowing a lysozyme to digest the cell wall [26]. In ECLFM1, putative holin (ORF_82) and phage lysozyme R (ORF_226) play a role in the lysis of bacterial cell walls. ORF_226 contains a Lyz-like domain (Table S1). The T4 lysozyme hydrolyzes the 1,4-beta linkages between N-acetyl-d-glucosamine and N-acetylmuramic acid in peptidoglycan heteropolymers of bacterial cells [38]. Further studies are required to determine the similarity in the mechanism of enzymatic activity of ORF_226 and the T4 lysozyme.

Comparative genomic studies using VICTOR and Mauve alignment revealed that ECLFM1 belonged to the *Tevenvirinae* subfamily of the *Straboviridae* family and was clustered with genus *Karamvirus*. It is most closely related to *Enterobacter* phage PG7, sharing a 96% similarity. Most ORFs in ECLFM1 show high similarity (90%) to the homologs in PG7; however, sequence variations were observed between these two phages, particularly in the regions encoding two tail fiber-associated proteins (ORF80 and ORF81) and several hypothetical proteins dispersed throughout the genome. Relative differences in ORF80 and ORF81 (63% and 73% similarity to PG7, respectively) associated with binding specificity to receptor proteins on the bacterial membrane cause variations in host range between the strains and varied EOP (Figure 2), as discussed in previous reports [39]. 

Similar to certain *Karamviruses* shown in Figure 10, ECLFM1 has a cluster of tRNA genes. The location and arrangement of tRNAs in ECLFM1 closely resemble those in PG7, with one significant difference: PG7 contains 20 tRNAs, whereas ECLFM1 has only 19 tRNAs, lacking one tRNA-Met. Additionally, one tRNA-Glx has been replaced by tRNA-Gln, and two tRNA-Asx have been replaced by tRNA-Asp and tRNA-Asn, respectively, in ECLFM1. The presence of tRNA genes is related to codon usage; a particular tRNA gene may also be favored because the phage utilizes the corresponding amino acid more frequently than its host. In such cases, the presence of the specific tRNA gene ensures an adequate supply of that particular amino acid during phage replication and protein synthesis. Overall, the presence of tRNA genes in phage genomes reflects phage adaptation in efficiently translating their genetic information and optimizing protein synthesis using codons and amino acids that are advantageous for their replication within the host cell. The diverse repertoire of tRNAs in ECLFM1 may also contribute to efficient phage production, as seen in previous reports [40]. A coliphage AR1 genome study found that tRNA genes were specifically related to optimal codon usage for the expression of late genes encoding structural proteins to increase both phage protein synthesis rate and burst size [41,42]. The presence of tRNAs is possibly relevant to lytic effects, broad host range, and enhancing phage fitness under various environments and external stress [40,43]. In ECLFM1, the tRNA genes cluster located near the start of the structural protein genes suggests their potential involvement in optimizing protein synthesis during phage replication. This positioning allows for the efficient translation of structural protein genes, which are crucial for phage particle assembly. The measured burst size of 75 PFU/infected cell at a low MOI of 0.0001 indicates a high level of phage production within infected cells. This high burst size suggests that ECLFM1 has evolved mechanisms to maximize its replication and the release of progeny phage particles. However, further investigation is required to confirm the specific role of the tRNAs and their impact on the overall efficiency of ECLFM1 replication. Furthermore, retention of ECLFM1 activity under normal conditions and loss of activity under extreme conditions (pH < 3 and >11, Tm > 65 °C, UV exposure for more than 1 h) suggests its stability and robustness, which is important for phage survival and effectiveness as a therapeutic agent.

One of the strengths of our study is that we successfully demonstrated the lytic effectiveness of the ECLFM1 phage and its ability to effectively treat bacterial infections in animals. This finding highlights the potential of this phage as a treatment option for infections caused by *E. cloacae* resistant to meropenem and colistin. Based on these promising results, further investigation of the therapeutic potential of this phage in treating drug-resistant bacterial infections should be performed.

## 4. Materials and Methods

### 4.1. Bacterial Strains and Culture Conditions

A total of 123 clinical strains of *E. cloacae* were provided by the Department of Medical Research, Hualien Tzu Chi Hospital, Buddhist Tzu Chi Medical Foundation, Hualien, Taiwan. Additional clinical isolates were included for phage host range testing, including 15 *E*. *coli*, 10 *Pseudomonas aeruginosa*, 8 *K*. *pneumoniae*, and 12 *Acinetobacter baumannii* strains. All bacterial strains were cultured on LB broth or agar (LA) at 37 °C under aerobic conditions. The bacterial lawn was established using the overlay agar method with 0.7% agar [44].

### 4.2. Phage Isolation and Purification

The *E. cloacae* phage used in this study was isolated from sewage samples collected around Tzu Chi Hospital in Hualien, Taiwan. The host strain used for isolation was ECL73134, a clinically isolated carbapenem-resistant *E. cloacae* strain carrying the *bla*_IMP-8_ gene. The sewage sample was processed via centrifugation at 8000× *g* for 10 min at room temperature, following filtration using a 0.45-μm syringe filter to remove large particulates and bacteria. Lytic phage presence was confirmed via observing bacterial lawn clearance using spot tests or a double-layer agar assay.

For CsCl gradient purification, isopycnic centrifugation was performed. A high titer phage lysate (10^12^ PFU) was precipitated via centrifugation at 15,000× *g* for 2 h at 4 °C using an Avanti JXN-26 centrifuge with a JA-25.50 rotor (Beckman Coulter Inc., Brea, CA, USA). The resulting phage pellet was suspended in sodium chloride-magnesium sulfate (SM) buffer (0.05 M Tris-HCl, pH 7.5, containing 0.1 M sodium chloride, 0.008 M MgSO_4_•7H_2_O, and 0.01% gelatin). This phage suspension was layered onto a CsCl block gradient with densities of 1.7, 1.5, 1.45, and 1.3 g/mL and centrifuged at 25,000 rpm for 3 h at 4 °C using an SW 41 Ti rotor in an Optima XPN-100 ultracentrifuge (Beckman Coulter). The banded phage particles were collected, dialyzed at 4 °C in SM buffer for 24 h, and stored at 4 °C for further use [44,45].

### 4.3. Morphological Observation Using TEM

A negatively stained sample was used to evaluate phage morphology using TEM. Ten microliters of dialyzed phage (ca. 10^10^ PFU) were spotted on the formvar-coated copper grid (300 mesh copper grids) and stained with 2% uranyl acetate. Morphological characteristics were observed under an H-7500 TEM (Hitachi, Tokyo, Japan) at an acceleration voltage of 80 kV with a CDD camera.

### 4.4. Host Range Determination and EOP of Phage

To determine the host range of the phage, a modified spot assay was performed as previously described [46]. Bacterial cultures in the mid-log phase (200 μL) were mixed with LB agar (5 mL) containing 0.7% agar and poured onto LA plates. After allowing the mixture to dry for 5 min at room temperature, 5 μL of the phage suspension (approximately 10^8^ PFU) was spotted on the agar surface. The plates were then incubated at 37 °C, and were examined after 16 h for the presence of cleared zones, indicating bacterial lysis caused by the phage. Clear zone formation at the spot indicated that the bacterial strain was sensitive to the phage and susceptible to lysis.

To determine phage lytic efficiency against susceptible strains, an EOP assay was performed as previously described [47]. Briefly, 100 μL of phage suspension (1 × 10^6^ PFU/mL) was mixed with 300 μL of mid-log phase culture of the tested bacteria and incubated for 10 min to allow phage adsorption to the host. The mixture was then added to 5 mL of soft agar (0.7% *w*/*v*) and poured onto LA plates. After solidification, the plates were incubated at 37 °C for 18 h. The plaques (clear zones) formed on each plate were counted, representing the infective phage particles. The relative EOP was calculated as the ratio of the average number of plaques (PFUs) on the tested bacteria and those on the host strain. The EOP values were categorized as high (EOP ≥ 0.5), moderate (0.1 ≤ EOP < 0.5), low (0.001 < EOP < 0.1), or inefficient (EOP ≤ 0.001) against the target strain, indicating phage efficiency in lysing the specific bacterial strain.

### 4.5. One-Step Growth and Adsorption Efficiency of Phage

To generate the one-step growth curve, a modified version of a previously reported method was followed [44]. A culture of the host bacteria (5 mL) was grown until the OD_600_ reached 0.6 to 0.8. Subsequently, the bacterial cells (1 mL, approximately 1.0 × 10^8^ CFU) were pelleted via centrifugation (8000× *g*, 5 min, room temperature), resuspended in 0.9 mL of SM buffer, and mixed with 0.1 mL of a phage suspension (1.0 × 10^5^ PFU/mL). The mixture was incubated on ice for 30 min. After centrifugation (8000× *g*, 2 min, room temperature), the supernatant containing free phage was removed and the pellet was suspended in 15 mL of LB medium. The mixture was then incubated at 37 °C with shaking at 200 rpm. Samples were collected at 5-min intervals up to 35 min, and phage titer was determined using the double-layer agar technique. The burst size was calculated as the ratio of the final count of liberated phage particles to the initial count of infected bacterial cells during the latent period [48].

Phage adsorption analysis was conducted as previously described [44]. Briefly, bacterial cells were infected with the phage at a MOI of 0.001 and incubated at 37 °C with shaking. Samples (100 µL) were collected after 0, 2, 4, 6, 8, and 10 min. The aliquots were then centrifuged at 12,000 rpm for 5 min, and a double-layer agar experiment was performed with the supernatants to determine the number of unadsorbed phages present. The phage adsorption efficiency (%) was calculated using the following equation: (initial titer of phage-titer of unadsorbed phages present in the supernatant)/initial titer of phage, multiplied by 100.

### 4.6. Bacteriolytic Characteristic of the Phage

In vitro, the bacteriolytic effect of phage was assessed using the microtiter plate liquid assay with a minor modification [49]. In brief, the host bacterial inoculum (~10^8^ CFU/mL) was adjusted via 100-fold dilution of overnight culture in LB and refreshing to an OD_600_ of ~0.3. Phage lysates were titered and adjusted to concentrations of 10^5^,10^6^, 10^7^, 10^8^, and 10^9^ PFU/mL with SM buffer. For each assay, 180 µL of adjusted bacterial inocula in LB were mixed with 20 µL of phage lysates with different doses in sterile, untreated Falcon^®^ 96-well transparent plates to achieve a final MOI of 0.001, 0.01, 0.1, 1, and 10, respectively. The plates were incubated at 37 °C with double orbital shaking, and growth was monitored via OD_600_ measurement at 1-h intervals for 10 h using a microtiter plate reader (Clariostar Plus, BMG Labtech, Offenburg, Germany), which resulted in 11 total time points including the initial (time 0) measurement. Growth curves were obtained via plotting OD after baseline adjustment against time. All assays were performed with three biological replicates.

### 4.7. Influence of the External Factors on Phage Infectivity

Phage infectivity tests were performed as described by Jurczak-Kurek et al. with a minor modification [50]. To determine the infection activity of phage lysate, the following external factors were tested: temperature (4, 37, 55, and 65 °C) and pH (3, 5, 7, 9, and 11). The phage lysate was diluted with LB (at the volume proportion 1:9) and incubated under the conditions described. The mixture was then withdrawn shortly, and serial 10-fold dilutions were used for double-layer agar plating. Phages without any treatment were the control. After overnight incubation at 37 °C, the percentage of remaining plaque-forming phages was calculated.

### 4.8. Phage DNA Extraction

To extract phage DNA, the phage lysate was initially treated with DNase I (1 U/μL; Thermo Fisher Scientific, Waltham, MA, USA) and RNase A (5 μg/μL; Thermo Fisher Scientific) at 37 °C for 30 min to degrade bacterial nucleic acids. Subsequently, a mixture containing 10 μg/mL proteinase K, 0.5 M EDTA, and 10% SDS was added for 3 h at 55 °C and inactive by 70 °C to disrupt the viral capsid and inactivate DNase I and RNase A. The sample was then subjected to phenol-chloroform extraction twice. The phage DNA pellet was precipitated via centrifugation at 12,000 rpm for 30 min using 95% alcohol, and washed with 75% ethanol. The pellet was then resuspended in 30 μL of Tris-EDTA (TE) buffer [51]. Purity and concentration of the extracted DNA were evaluated using nanodrop measurements performed on a NanoDrop™ 2000C Spectrophotometer (Thermo Fisher Scientific).

### 4.9. DNA Sequencing and Genome Analysis of Phage

The isolated phage genomic DNA (~5 μg) was sent to Allbio Life Co., Ltd. (Taichung, Taiwan) for genome sequencing, quality assessment of sequence reads, and de novo assembly. The phage DNA was fragmented down to a length of approximately 500 bp using the Covaris ultrasonic crusher (Covaris, LLC., Woburn, MA, USA), and end repair was performed before sticky end generation by adding base A to the 3′ end. Electroporation was employed for target fragment recovery, following PCR amplification of the DNA fragments flanked by adapters. The PCR products were cleaned and validated using Bioanalyzer (Agilent, Santa Clara, CA, USA). The qualified libraries were sequenced (paired-end 150) on the HiseqXten/Novaseq/MGI2000 System (Illumina, San Diego, CA, USA). The open reading frame was annotated using the RAST annotation server web [52]. Automatic annotation was manually reviewed using the BLASTp algorithm against RefSeq proteins deposited in the GenBank database [53]. The whole-genome phylogenetic tree was constructed via tree building online resource (VICTOR) [54]. tRNAs were predicted using tRNAscan-SE2.02 [55]. Virulence factors and drug resistance of phage genome were compared using VirulenceFinder 2.0 and ResFinder 1.4 [56]. Alignments of the phage genomes were visualized via the Easyfig program [57]. A phylogenetic tree of the terminase large subunit (TerL) was generated in MEGA 11 using the neighbor joining approach and 1000 bootstrap replications [58]. The ECLFM1 genome sequence with annotations was deposited in the GenBank database under accession number OQ411233.

### 4.10. Phage Structural Protein Analysis via Liquid Chromatography Coupled to Tandem Mass Spectrometry

To analyze virion proteins, CsCl-purified phage particles were mixed with a lysis buffer containing 62.5 mM Tris-HCl (pH 6.8), 5% 2-mercaptoethanol, 2% sodium dodecyl sulfate (SDS), 10% glycerol, and 0.01% bromophenol blue. The mixture was boiled for 10 min and separated via 12% SDS-PAGE. Protein bands were visualized using Coomassie blue staining. Proteins of interest were excised from the stained gels and subjected to in-gel digestion. The excised gel pieces were first reduced and then alkylated. The proteins were then digested with trypsin (Promega, Madison, WI, USA), and the resulting peptides were extracted from the gel pieces. The peptide mixture was analyzed using an UltiMate 3000 RSLCnano system coupled to a Q Exactive mass spectrometer (Thermo Fisher Scientific). The raw data obtained from the mass spectrometer were searched against a local database consisting of all possible peptide spectra deduced from the ECLFM1 genome sequence.

### 4.11. Evaluation of Phage Effectiveness against E. cloacae-Infected Zebrafish

In accordance with established procedures, the Tzu Chi University Fish Core facility followed the standard methods to maintain and produce the zebrafish (*Danio rerio*) employed in this study. Zebrafish mixed with males and females were housed in 9 L tanks at 28 °C with a 14 h light/10 h dark cycle. All protocols employed in this study were in accordance with the rules and regulations of and approved by the Tzu Chi University Institutional Animal Care and Use Committee (IACUC, approval no. 111091). The experimental procedures were carried out as reported in previously published articles [59]. Prior to phage treatment, the lethal dose (LD_50_) of *E. cloacae* for zebrafish was determined. Adult disease-free zebrafish (3–3.5 cm-sized) were injected with different doses of wild-type *E. cloacae* strain (3 × 10^6^, 3 × 10^7^, and 3 × 10^8^ CFU) via the cloaca using an insulin needle. Each fish in the experimental group (*n* = 8) was injected with 20 μL of purified phage (3 × 10^9^ PFU), and 20 μL of SM buffer was injected to the control group for phage safety testing. Further, to examine the impact of phage treatment on *E. cloacae*-infected zebrafish, the phage was administered into the zebrafish cloaca after 30 min of *E. cloacae* injection. Before injection, fish were anesthetized with 0.2% tricaine. After injection, injected fish were transferred into a separate water tank, and their survival rate was monitored at room temperature via observation every 6 h for 24 h. Any behavioral changes, such as jumping from the tank, slow or fast movement, gulping for air, or moving over the beaker base, were noted and considered a potential sign of morbidity.

### 4.12. Statistical Analysis

Statistical analysis of significance was conducted using GraphPad Prism 9 software version 9.0.0. One-way analysis of variance and Dunnett’s multiple comparison tests were performed to assess the overall significance and compare specific groups, respectively. A significance level of *p* ≤ 0.05 was considered statistically significant. All experiments were repeated in triplicate to ensure reliability and consistency of the results.

## 5. Conclusions

The ECLFM1 phage is a promising candidate for phage therapy against carbapenem-resistant *E. cloacae* infections. The findings of this study highlight the biological characteristics, stability, and genomic features of ECLFM1, supporting its potential as an alternative treatment option for carbapenem-resistant *E. cloacae* infections. Further research is required to assess its therapeutic efficacy, safety, and clinical applicability against multidrug-resistant *E. cloacae* infections

## Data Availability

The data presented in this study are available on request from the corresponding author.

## Supplementary Materials

The following supporting information can be downloaded at: https://www.mdpi.com/article/10.3390/ijms25020854/s1.
