# Supplementary material for: Therapeutic Potential of a Novel Lytic Phage, vB_EclM_ECLFM1, against Carbapenem-Resistant Enterobacter cloacae
Source: Int J Mol Sci. 2024 Jan 10;25(2):854. doi: 10.3390/ijms25020854 (PMC10815064; doi:10.3390/ijms25020854)
Supplement: Supplementary file 1 [file ijms-25-00854-s001.zip › ijms-2759566-supplementary.pdf]

Table S1: Functional genomic annotation of phage ECLFM-1

ORF	Strand	Function	Related protein (Best Match)	Domain	Amino acid length
repeat-1	+	repeat region			
1	-	hypothetical protein	hypothetical protein prasa_gp213 [Enterobacter phage prasa_myo]		181
2	-	RNA ligase	RNA ligase 2 [Enterobacter phage CC31]	Adenylation_ DNA_ligase_ like superfamily	334
3	-	hypothetical protein	hypothetical protein CG98_gp247 [Enterobacter phage PG7]		82
4	-	hypothetical protein	hypothetical protein KNT83_gp162 [Enterobacter phage myPSH1140]		117
5	-	hypothetical protein	putative DNA primase-helicase subunit [Klebsiella phage vB_KaeM_KaAlpha]	DUF2774 superfamily	80
6	-	capsid and scaffold protein	putative outer capsid [Klebsiella phage vB_KaeM_KaAlpha]		373
7	-	hypothetical protein	putative inhibitor of prohead protease [Klebsiella phage vB_KaeM_KaAlpha]		239
8	+	Phage DNA helicase	putative split helicase [Klebsiella phage vB_KaeM_KaAlpha]	uvsW superfamily	500
9	+	UvsW.1 conserved hypothetical protein	putative split helicase [Enterobacter phage PG7]	UvsW superfamily	79
10	-	hypothetical protein	hypothetical protein KAALPHA_227 [Klebsiella phage vB_KaeM_KaAlpha]	DUF2685 superfamily	55
11	-	Phage protein	hypothetical protein KNT70_gp222 [Cronobacter phage Pet-CM3-4]		69

12	-	Single stranded DNA-binding protein, phage-associated	Single stranded DNA-binding protein, phage-associated [Cronobacter phage Pet-CM3-4]	UvsY superfamily	137
13	-	Phage baseplate wedge subunit (T4-like gp25)	putative baseplate wedge subunit [Klebsiella phage vB_KaeM_KaAlpha]	GPW_gp25 superfamily	132
14	-	Phage baseplate hub subunit (T4-like gp26)	putative baseplate hub subunit [Klebsiella phage vB_KaeM_KaAlpha]	T4_baseplate superfamily	209
15	+	Phage baseplate	baseplate hub assembly protein [Enterobacter phage PG7]	T4_baseplate superfamily	250
16	+	baseplate hub subunit	baseplate hub subunit [Enterobacter phage vB-EclM_KMB17]	27 superfamily	394
17	+	Phage baseplate hub	putative baseplate distal hub subunit [Klebsiella phage vB_KaeM_KaAlpha]	Phage_hub_GP28 superfamily	167
18	+	hypothetical protein	baseplate hub subunit tail length determinator [Enterobacter phage PG7]	COG5283 superfamily	586
19	+	Phage baseplate tail tube cap (T4-like gp48)	baseplate tail tube cap [Enterobacter phage vB-EclM_KMB20]	T4_tail_cap superfamily	349
20	+	tail assembly protein	putative baseplate tail tube initiator [Klebsiella phage vB_KaeM_KaAlpha]	19 superfamily	320
21	-	hypothetical protein	hypothetical protein CC31p198 [Enterobacter phage CC31]	DUF5498 superfamily	96
22	-	hypothetical protein	hypothetical protein prasa_gp234 [Enterobacter phage prasa_myo]	alt superfamily	709
23	-	hypothetical protein	hypothetical protein CC31p200 [Enterobacter phage CC31]		72
24	-	DNA ligase, phage-	DNA ligase [Enterobacter phage PG7]	30 superfamily	481

as

25	-	hypothetical protein	hypothetical protein [Enterobacter phage vB-EclM_KMB20]		76
26	-	hypothetical protein	hypothetical protein KAALPHA_242 [Klebsiella phage vB_KaeM_KaAlpha]	30.2 superfamily	289
27	-	hypothetical protein	hypothetical protein CC31p204 [Enterobacter phage CC31]		58
28	-	hypothetical protein	hypothetical protein CG98_gp222 [Enterobacter phage PG7]		67
29	-	hypothetical protein	hypothetical protein CG98_gp221 [Enterobacter phage PG7]		55
30	-	hypothetical protein	hypothetical protein KNT70_gp203 [Cronobacter phage Pet-CM3-4]		156
31	-	hypothetical protein	hypothetical protein KAALPHA_248 [Klebsiella phage vB_KaeM_KaAlpha]	Phage_T4_ Gp30_7 superfamily	120
32	-	hypothetical protein	hypothetical protein KAALPHA_249 [Klebsiella phage vB_KaeM_KaAlpha]		173
33	-	hypothetical protein	hypothetical protein CG98_gp217 [Enterobacter phage PG7]	Phage_30_8 superfamily	124
34	-	hypothetical protein	hypothetical protein KAALPHA_251 [Klebsiella phage vB_KaeM_KaAlpha]		73
35	-	hypothetical protein	hypothetical protein KNT70_gp198 [Cronobacter phage Pet-CM3-4]		59
36	-	lysis inhibition accessory protein, rapid lysis phenotype	lysis inhibition accessory protein, rapid lysis phenotype [Enterobacter phage PG7]		84
37	-	Phage head assembly chaperone protein (T4-	capsid assembly chaperone [Enterobacter phage vB-EclM_KMB20]		107

li

38	-	hypothetical protein	hypothetical protein KNT70_gp195 [Cronobacter phage Pet-CM3-4]	WYL_2 superfamily	109
39	-	dCMP deaminase (EC 3.5.4.12)	deoxycytidylate deaminase [Enterobacter phage PG7]	cytidine_ deaminase-like superfamily	188
40	-	hypothetical protein	hypothetical protein KNT83_gp195 [Enterobacter phage myPSH1140]		114
41	-	hypothetical protein	hypothetical protein [Enterobacter phage vB-EclM_KMB19]		76
42	-	hypothetical protein	hypothetical protein KAALPHA_260 [Klebsiella phage vB_KaeM_KaAlpha]		97
43	-	hypothetical protein	hypothetical protein CG98_gp207 [Enterobacter phage PG7]		71
44	-	hypothetical protein	hypothetical protein CG98_gp206 [Enterobacter phage PG7]		63
45	-	3'-phosphatase, 5'-polynucleotide kinase, phage-associated	polynucleotide kinase [Enterobacter phage PG7]	pseT superfamily	295
46	-	hypothetical protein	hypothetical protein CG98_gp204 [Enterobacter phage PG7]		119
47	-	hypothetical protein	hypothetical protein CG98_gp203 [Enterobacter phage PG7]		70
48	-	hypothetical protein	hypothetical protein CG98_gp202 [Enterobacter phage PG7]		159
49	-	hypothetical protein	hypothetical protein CC31p227 [Enterobacter phage CC31]		32
50	-	hypothetical protein	hypothetical protein CG98_gp200 [Enterobacter phage		100

51	-	predicted membrane protein	predicted membrane protein [Enterobacter phage CC31]	PHA02141 superfamily	118
52	-	inhibitor of host transcription	inhibitor of host transcription [Enterobacter phage PG7]	ALP superfamily	168
53	-	RNA ligase	RNA ligase [Enterobacter phage fGh-Ecl04]	RNA_lig_T4_1 superfamily	376
54	-	endonuclease	putative endonuclease II [Klebsiella phage vB_KaeM_KaAlpha]	GIY-YIG_SF superfamily	137
55	-	Ribonucleotide reductase of class Ia (aerobic), beta subunit (EC 1.17.4.1)	ribonucleotide reductase of class Ia (aerobic), beta subunit [Enterobacter phage prasa_myo]	Ferritin_like superfamily	404
56	-	hypothetical protein	hypothetical protein KNT70_gp177 [Cronobacter phage Pet-CM3-4]		73
57	-	hypothetical protein	hypothetical protein KAALPHA_275 [Klebsiella phage vB_KaeM_KaAlpha]		58
58	-	Ribonucleotide reductase of class Ia (aerobic), alpha subunit (EC 1.17.4.1)	ribonucleotide reductase of class Ia, alpha subunit [Enterobacter phage vB-EclM_KMB19]	nrdA superfamily	751
59	-	Phage protein (ACLAME 596)	DUF4326 domain-containing protein [Enterobacter phage CC31]	DUF4326 superfamily	103
60	-	hypothetical protein	hypothetical protein CC31p236 [Enterobacter phage CC31]		77
61	-	dTMP thymidylate synthase	dTMP thymidylate synthase [Enterobacter phage fGh-Ecl01]		62
62	-	Thymidylate synthase (EC 2.1.1.45)	dTMP thymidylate synthase [Enterobacter phage fGh-Ecl01]	TS_Pyrimidine_HMase superfamily	286

63	-	hypothetical protein	hypothetical protein CG98_gp189 [Enterobacter phage PG7]		115
64	-	hypothetical protein	hypothetical protein KNT70_gp171 [Cronobacter phage Pet-CM3-4]		106
65	-	dihydrofolate reductase	Dihydrofolate reductase [Cronobacter phage Pet-CM3-4]	DHFR superfamily	197
66	-	hypothetical protein	hypothetical protein CG98_gp186 [Enterobacter phage PG7]		70
67	-	hypothetical protein	hypothetical protein KAALPHA_286 [Klebsiella phage vB_KaeM_KaAlpha]		107
68	-	hypothetical protein	hypothetical protein CG98_gp182 [Enterobacter phage PG7]		94
69	-	hypothetical protein	hypothetical protein CG98_gp181 [Enterobacter phage PG7]		47
70	-	hypothetical protein	hypothetical protein KAALPHA_289 [Klebsiella phage vB_KaeM_KaAlpha]		90
71	-	hypothetical protein	hypothetical protein CG98_gp179 [Enterobacter phage PG7]		74
72	-	Single stranded DNA-binding protein, phage-associated	gp32 single-stranded DNA binding protein [Enterobacter phage CC31]	gp32 superfamily	306
73	-	Phage DNA helicase loader	59 protein [Enterobacter phage CC31]	59 superfamily	217
74	-	gp33 late promoter transcription accessory protein	gp33 late promoter transcription accessory protein [Enterobacter phage CC31]	Trans_coact superfamily	99
75	-	dsDNA binding protein, late tr	double-stranded DNA binding protein [Enterobacter phage PG7]	Phage_DsbA superfamily	91

76	-	Phage ribonuclease H (EC 3.1.26.4)	RnaseH [Enterobacter phage PG7]	rnh superfamily	307
77	+	long tail fiber proximal subunit	putative long tail fiber proximal subunit [Klebsiella phage vB_KaeM_KaAlpha]	34 superfamily	1264
78	+	hinge connector of long tail fiber proximal connector	putative hinge connector of long tail fiber [Klebsiella phage vB_KaeM_KaAlpha]	PANDER_like superfamily	382
79	+	Phage tail fiber protein (long tail fiber) (gp36)	Hinge connector of long tail fiber distal connector [Cronobacter phage Pet-CM3-4]		217
80	+	hypothetical protein	long tail fiber, distal subunit [Enterobacter phage vB_EclM_CIP9]	Peptidase_S74_CIMCD superfamily	847
81	+	Phage tail fiber protein (long tail fiber) (gp38)	receptor recognizing protein [Enterobacter phage ENC9]	GP38 superfamily	259
82	+	putative holin	holin [Enterobacter phage vB-EclM_KMB19]	Phage_holin_T superfamily	218
83	-	Anti-sigma factor	anti-sigma 70 protein [Enterobacter phage PG7]	AsiA superfamily	90
84	-	hypothetical protein	hypothetical protein CC31p261 [Enterobacter phage CC31]		85
85	-	hypothetical protein	hypothetical protein CG98_gp165 [Enterobacter phage PG7]		116
86	-	hypothetical protein	hypothetical protein CC31p263 [Enterobacter phage CC31]		92
87	-	hypothetical protein	hypothetical protein CC31p264 [Enterobacter phage CC31]		40
88	-	hypothetical protein	hypothetical protein KNT83_gp231 [Enterobacter phage myPSH1140]		95
89	-	hypothetical protein	hypothetical protein HWC54_gp157 [Klebsiella phage		170

90	-	hypothetical protein	hypothetical protein CG98_gp161 [Enterobacter phage PG7]		134
91	-	activator of middle period transcription	Activator of middle period transcription [Cronobacter phage Pet-CM3-4]	MotA_activ superfamily	214
92	-	hypothetical protein	hypothetical protein [Enterobacter phage vB-EclM_KMB17]		90
93	-	hypothetical protein	hypothetical protein [Enterobacter phage vB-EclM_KMB17]		87
94	-	DNA topoisomerase	DNA topoisomerase [Enterobacter phage vB-EclM_KMB17]	TOP4c superfamily	459
95	-	hypothetical protein	hypothetical protein CG98_gp156 [Enterobacter phage PG7]		44
96	-	hypothetical protein	hypothetical protein CG98_gp155 [Enterobacter phage PG7]		60
97	-	nucleoid disruption protein	nucleoid disruption protein [Enterobacter phage PG7]	Phage_T4_Ndd superfamily	148
98	-	hypothetical protein	hypothetical protein KNT70_gp135 [Cronobacter phage Pet-CM3-4]		69
99	-	hypothetical protein	hypothetical protein KNT70_gp134 [Cronobacter phage Pet-CM3-4]		39
100	-	Phage endonuclease	putative DNA endonuclease IV [Klebsiella phage vB_KaeM_KaAlpha]		186
101	-	hypothetical protein	putative rIIB lysis inhibitor [Klebsiella phage vB_KaeM_KaAlpha]		312
102	-	hypothetical protein	putative rllA lysis inhibitor [Klebsiella phage vB_KaeM_KaAlpha]	HATPase superfamily	737
103	-	hypothetical protein	hypothetical protein CG98_gp002 [Enterobacter phage		68

104	-	hypothetical protein	hypothetical protein CC31p003 [Enterobacter phage CC31]		95
105	-	hypothetical protein	hypothetical protein CC31p004 [Enterobacter phage CC31]	Phage_GPD superfamily	51
106	-	Phage DNA topoisomerase large subunit (EC 5.99.1.3)	DNA topoisomerase II [Enterobacter phage CC31]	39 superfamily	613
107	-	hypothetical protein	hypothetical protein CG98_gp005 [Enterobacter phage PG7]		128
108	-	hypothetical protein	putative head outer capsid protein [Klebsiella phage vB_KaeM_KaAlpha]	18 superfamily	511
109	-	hypothetical protein	hypothetical protein KNT70_gp007 [Cronobacter phage Pet-CM3-4]		86
110	-	hypothetical protein	hypothetical protein CG98_gp008 [Enterobacter phage PG7]		31
111	-	conserved putative regulatory protein, FmdB family	conserved putative regulatory protein, FmdB family [Enterobacter phage CC31]	Zn-ribbon_8 superfamily	58
112	-	hypothetical protein	hypothetical protein [Escherichia coli]		111
113	-	hypothetical protein	hypothetical protein prasa_gp14 [Enterobacter phage prasa_myo]		225
114	-	modifier of suppressor tRNAs	modifier of suppressor tRNAs [Enterobacter phage vB-EclM_KMB20]		72
115	-	hypothetical protein	hypothetical protein CG98_gp014 [Enterobacter phage PG7]		145
116	-	exonuclease	T4-like phage DexA exonuclease A [Cronobacter phage Pet-CM3-4]	DnaQ_like_exo superfamily	229
117	-	hypothetical protein	hypothetical protein CG98_gp016 [Enterobacter phage		80

118	-	DNA helicase	DNA helicase [Enterobacter phage PG7]	DEAD-like_helicase_N superfamily	443
119	-	hypothetical protein	hypothetical protein KNT70_gp016 [Cronobacter phage Pet-CM3-4]		104
120	-	putative anti-sigma factor	Putative anti-sigma factor [Cronobacter phage Pet-CM3-4]		250
121	-	RNA polymerase ADP-ribosylase	ADP-ribosylase [Enterobacter phage PG7]		225
122	-	hypothetical protein	hypothetical protein CG98_gp021 [Enterobacter phage PG7]		69
123	-	hypothetical protein	hypothetical protein [Enterobacter phage vB-EclM_KMB20]		169
124	-	hypothetical protein	hypothetical protein KAALPHA_27 [Klebsiella phage vB_KaeM_KaAlpha]		130
125	-	hypothetical protein	hypothetical protein CC31p024 [Enterobacter phage CC31]		83
126	-	Phage head decoration protein (Soc)	small outer capsid protein [Enterobacter phage PG7]	Soc superfamily	78
127	-	hypothetical protein	hypothetical protein KNT70_gp024 [Cronobacter phage Pet-CM3-4]		121
128	-	dUTPase	dCTP pyrophosphatase [Enterobacter phage PG7]	NTP-PPase superfamily	172
129	-	hypothetical protein	hypothetical protein KAALPHA_32 [Klebsiella phage vB_KaeM_KaAlpha]		94
130	-	hypothetical protein	DNA primase / DNA helicase, phage-associated [Cronobacter phage Pet-CM3-4]	61 superfamily	341
131	-	hypothetical protein	hypothetical protein CG98_gp030 [Enterobacter phage	Gp23 superfamily	154

132	-	hypothetical protein	hypothetical protein CG98_gp031 [Enterobacter phage PG7]		62
133	-	hypothetical protein	hypothetical protein CG98_gp032 [Enterobacter phage PG7]		65
134	-	hypothetical protein	spackle periplasmic protein [Enterobacter phage PG7]		93
135	-	hypothetical protein	hypothetical protein KAALPHA_38 [Klebsiella phage vB_KaeM_KaAlpha]		91
136	-	Phage DNA primase/helicase	putative DNA primase/DNA helicase [Klebsiella phage vB_KaeM_KaAlpha]	41 superfamily	480
137	-	Phage capsid and scaffold	putative head vertex assembly chaperone [Klebsiella phage vB_KaeM_KaAlpha]	Phage_head_chap superfamily	122
138	-	recombination protein	putative RecA-like recombinase protein [Klebsiella phage vB_KaeM_KaAlpha]	P-loop_NTPase superfamily	387
139	-	beta glucosyl transferase	DNA beta-glucosyltransferase [Enterobacter phage KKP_3262]	Glycosyl transferase_GTB-type superfamily	358
140	-	glucosyl transferase	hypothetical protein CC31p040 [Enterobacter phage CC31]		276
141	-	hypothetical protein	Thymidylate synthase [Cronobacter phage Pet-CM3-4]	TS_Pyrimidine_HMase superfamily	245
142	-	hypothetical protein	hypothetical protein KAALPHA_46 [Klebsiella phage vB_KaeM_KaAlpha]		146
143	-	hypothetical protein	hypothetical protein CG98_gp042 [Enterobacter phage PG7]		74
144	-	DNA polymerase (EC 2.7.7.7), phage-as	DNA polymerase [Enterobacter phage fGh-Ecl04]	43 superfamily	902

145	-	gp43.1 hypothetical protein	hypothetical protein [Enterobacter phage vB-EclM_KMB17]		53
146	-	hypothetical protein	hypothetical protein [Enterobacter phage vB-EclM_KMB17]		66
147	-	Phage endoribonulcease translational repressor of early genes, regA	translational repressor RegA [Enterobacter phage CC31]	Translat_reg superfamily	121
148	-	Phage DNA polymerase clamp loader subunit Gp62	clamp loader small subunit [Enterobacter phage PG7]	Phage_clamp_A superfamily	187
149	-	Replication factor C small subunit / Phage DNA polymerase clamp loader subunit	clamp loader small subunit [Enterobacter phage fGh-Ecl04]	44 superfamily	319
150	-	Sliding clamp DNA polymerase accessory protein, phage associated	DNA polymerase [Enterobacter phage CC31]	45 superfamily	228
151	-	hypothetical protein	RNA polymerase binding protein [Enterobacter phage myPSH1140]	Phage_RpbA superfamily	138
152	-	hypothetical protein	hypothetical protein KAALPHA_56 [Klebsiella phage vB_KaeM_KaAlpha]	Gp45_2 superfamily	63
153	-	Phage recombination-related endonuclease Gp46	recombination-related endonuclease [Enterobacter phage vB-EclM_KMB20]	46 superfamily	562
154	-	Phage recombination-related endonuclease G	metallophosphoesterase [Enterobacter phage myPSH1140]	47 superfamily	340

155	-	hypothetical protein	hypothetical protein ACQ46_gp060 [Citrobacter phage Moon]		33
156	-	hypothetical protein	prohead core protein protease [Citrobacter phage Merlin]	Peptidase_S77 superfamily	61
157	-	hypothetical protein	hypothetical protein ACQ46_gp062 [Citrobacter phage Moon]		69
158	-	a-gt.4 hypothetical protein	hypothetical protein CPT_Merlin63 [Citrobacter phage Merlin]	DUF2654 superfamily	122
159	-	hypothetical protein	hypothetical protein CPT_Merlin64 [Citrobacter phage Merlin]	DUF5491 superfamily	42
160	-	T4-like phage RNA polymerase sigma factor for late transcription	RNA polymerase sigma factor [Enterobacter phage CC31]	55 superfamily	177
161	-	hypothetical protein	hypothetical protein KAALPHA_64 [Klebsiella phage vB_KaeM_KaAlpha]		98
162	-	hypothetical protein	hypothetical protein KAALPHA_65 [Klebsiella phage vB_KaeM_KaAlpha]		83
163	-	hypothetical protein	hypothetical protein CG98_gp061 [Enterobacter phage PG7]	DUF5495 superfamily	75
164	-	hypothetical protein	hypothetical protein KKP3262_000034 [Enterobacter phage KKP_3262]		87
165	-	hypothetical protein	hypothetical protein CG98_gp063 [Enterobacter phage PG7]		112
166	-	hypothetical protein	hypothetical protein KAALPHA_68 [Klebsiella phage vB_KaeM_KaAlpha]		88
167	-	glutaredoxin	glutaredoxin [Enterobacter phage CC31]	Thioredoxin_like superfamily	104
168	-	hypothetical protein	hypothetical protein CG98_gp066 [Enterobacter phage		91

169	-	hypothetical protein	hypothetical protein [Enterobacter phage vB-EclM_KMB20]		35
170	-	Ribonucleotide reductase of class III (anaerobic), activating protein (EC 1.97.1.4)	anaerobic ribonucleoside-triphosphate reductase activating protein [Cronobacter phage Pet-CM3-4]	NrdG superfamily	161
171	-	Ribonucleotide reductase of class III (anaerobic), large subunit (EC 1.17.4.2)	anaerobic NTP reductase large subunit [Enterobacter phage PG7]	RNR_PFL superfamily	609
172	-	gp49 recombinase endonuclease VII	recombination endonuclease VII [Enterobacter phage PG7]	49 superfamily	157
173	-	hypothetical protein	protease inhibitor [Enterobacter phage myPSH1140]	Inhibitor_I24 superfamily	152
174	-	hypothetical protein	hypothetical protein CG98_gp073 [Enterobacter phage PG7]		103
175	-	hypothetical protein	hypothetical protein CG98_gp074 [Enterobacter phage PG7]		57
176	-	thioredoxin	glutaredoxin [Enterobacter phage KKP_3262]	Thioredoxin_like superfamily	93
177	-	hypothetical protein	hypothetical protein CC31p076 [Enterobacter phage CC31]		73
178	-	hypothetical protein	hypothetical protein KAALPHA_81 [Klebsiella phage vB_KaeM_KaAlpha]		97
179	-	hypothetical protein	hypothetical protein KKP3262_000049 [Enterobacter phage KKP_3262]		111
180	-	hypothetical protein	hypothetical protein KAALPHA_83 [Klebsiella phage		151

181	-	predicted membrane protein	hypothetical protein CG98_gp080 [Enterobacter phage PG7]		141
182	-	hypothetical protein	hypothetical protein CG98_gp081 [Enterobacter phage PG7]		85
183	-	hypothetical protein	hypothetical protein KAALPHA_86 [Klebsiella phage vB_KaeM_KaAlpha]	PRK05771 superfamily	332
184	-	hypothetical protein	hypothetical protein KAALPHA_89 [Klebsiella phage vB_KaeM_KaAlpha]		257
185	-	hypothetical protein	hypothetical protein prasa_gp85 [Enterobacter phage prasa_myo]		122
186	-	hypothetical protein	hypothetical protein CG98_gp087 [Enterobacter phage PG7]		144
187	-	hypothetical protein	hypothetical protein CG98_gp088 [Enterobacter phage PG7]		71
188	-	hypothetical protein	hypothetical protein [Enterobacter phage vB-EclM_KMB19]		60
189	-	hypothetical protein	hypothetical protein [Enterobacter phage vB-EclM_KMB19]		155
190	-	hypothetical protein	hypothetical protein [Enterobacter phage vB-EclM_KMB19]		167
191	-	hypothetical protein	hypothetical protein [Enterobacter phage vB-EclM_KMB19]		113
192	-	hypothetical protein	hypothetical protein CG98_gp092 [Enterobacter phage PG7]	DUF5484 superfamily	48
193	-	thioredoxin	putative thioredoxin [Klebsiella phage vB_KaeM_KaAlpha]		325
194	-	hypothetical protein	hypothetical protein CG98_gp097 [Enterobacter phage PG7]		94
19	-	hypothetical protein	hypothetical protein KKP3262_000067 [Enterobacter phage KKP_	Nuc-transf superfamily	336

196	-	hypothetical protein	hypothetical protein KNT83_gp081 [Enterobacter phage myPSH1140]		141
197	-	hypothetical protein	hypothetical protein KAALPHA_103 [Klebsiella phage vB_KaeM_KaAlpha]		184
198	-	hypothetical protein	hypothetical protein CG98_gp101 [Enterobacter phage PG7]		73
199	-	hypothetical protein	hypothetical protein CG98_gp102 [Enterobacter phage PG7]		150
200	-	hypothetical protein	hypothetical protein KAALPHA_106 [Klebsiella phage vB_KaeM_KaAlpha]		81
201	-	hypothetical protein	hypothetical protein CG98_gp104 [Enterobacter phage PG7]		92
202	-	hypothetical protein	hypothetical protein CG98_gp105 [Enterobacter phage PG7]		68
203	-	hypothetical protein	hypothetical protein KNT83_gp087 [Enterobacter phage myPSH1140]		95
204	-	Uncharacterized zinc-finger containing protein YbiI	conserved hypothetical bacterial protein [Enterobacter phage CC31]	zf-dskA_traR superfamily	89
205	-	hypothetical protein	hypothetical protein KNT70_gp096 [Cronobacter phage Pet-CM3-4]		152
206	-	Phage protein	hypothetical protein KNT70_gp097 [Cronobacter phage Pet-CM3-4]	DUF5856 superfamily	130
207	-	lysis inhibition regulator membrane protein	lysis inhibition regulator [Enterobacter phage PG7]	PHA02054 superfamily	91
208	-	hypothetical protein	hypothetical protein CC31p111 [Enterobacter phage		70

209	-	hypothetical protein	hypothetical protein KNT83_gp093 [Enterobacter phage myPSH1140]		127
210	-	hypothetical protein	hypothetical protein CG98_gp114 [Enterobacter phage PG7]		43
211	-	hypothetical protein	hypothetical protein CC31p115 [Enterobacter phage CC31]		54
212	-	hypothetical protein	hypothetical protein [Enterobacter phage vB-EclM_KMB19]		41
213	-	thymidine kinase (EC 2.7.1.21)	thymidine kinase [Enterobacter phage CC31]	TK superfamily	194
214	-	hypothetical protein	hypothetical protein [Enterobacter phage vB-EclM_KMB17]	Macro_SF superfamily	151
215	-	hypothetical protein	hypothetical protein CG98_gp117 [Enterobacter phage PG7]		139
216	-	valyl-tRNA synthetase modifier	valyl-tRNA synthetase modifier [Enterobacter phage CC31]		108
217	-	hypothetical protein	hypothetical protein CG98_gp119 [Enterobacter phage PG7]	Lyz-like superfamily	182
218	-	Phage endonuclease	site-specific RNA endonuclease [Enterobacter phage CC31]	REGB_T4 superfamily	154
219	-	hypothetical protein	hypothetical protein CG98_gp121 [Enterobacter phage PG7]		76
220	-	Phage protein	hypothetical protein CG98_gp122 [Enterobacter phage PG7]		93
221	-	Autonomous glycyl radical cofactor	autonomous glycyl radical cofactor GrcA [Enterobacter phage PG7]	RNR_PFL superfamily	120
222	-	hypothetical protein	hypothetical protein [Enterobacter phage vB-EclM_KMB17]		50
223	-	Phage protein	hypothetical protein		139

KNT8

			224[Enterobacter phage my225PSH1140]		
224	-	hypothetical protein	Hypoth226etical protein CG98_gp125 [Enterobacter phage PG7]		171
225	-	hypothetical protein	hypothetical protein CG98_gp127 [Enterobacter phage PG7]		98
226	-	Phage lysozyme R (EC 3.2.1.17)	glycoside hydrolase family protein [Cronobacter phage Pet-CM3-4]	Lyz-like superfamily	164
227	-	Nudix hydrolase, phage-associated	nudix hydrolase [Enterobacter phage CC31]	Nudix_Hydrolase superfamily	159
228	-	hypothetical protein	hypothetical protein KNT70_gp117 [Cronobacter phage Pet-CM3-4]		76
229	-	hypothetical protein	hypothetical protein KKP3262_000098 [Enterobacter phage KKP_3262]	PHA02152 superfamily	103
230	-	hypothetical protein	hypothetical protein KAALPHA_138 [Klebsiella phage vB_KaeM_KaAlpha]		120
231	-	hypothetical protein	hypothetical protein KKP3262_000100 [Enterobacter phage KKP_3262]		112
232	-	hypothetical protein	hypothetical protein [Enterobacter phage vB-EclM_KMB17]		112
233	-	hypothetical protein	hypothetical protein CG98_gp135 [Enterobacter phage PG7]		124
234	-	hypothetical protein	hypothetical protein CC31p140 [Enterobacter phage CC31]		305
235	-	hypothetical protein	hypothetical protein CG98_gp137 [Enterobacter phage PG7]		117
236	-	hypothetical protein	hypothetical protein CG98_gp138 [Enterobacter phage	PHA02102 superfamily	77

237	-	Phage protein (ACLAME 1472)	hypothetical protein CG98_gp139 [Enterobacter phage PG7]		94
238	-	hypothetical protein	hypothetical protein prasa_gp140 [Enterobacter phage prasa_myo]		221
239	-	hypothetical protein	hypothetical protein KNT83_gp116 [Enterobacter phage myPSH1140]	DUF4810 superfamily	200
240	-	hypothetical protein	hypothetical protein prasa_gp142 [Enterobacter phage prasa_myo]		78
241	-	hypothetical protein	hypothetical protein [Enterobacter phage vB-EclM_KMB20]		66
242	-	hypothetical protein	hypothetical protein KNT70_gp128 [Cronobacter phage Pet-CM3-4]		86
243	-	hypothetical protein	hypothetical protein prasa_gp146 [Enterobacter phage prasa_myo]		218
244	-	hypothetical protein	hypothetical protein CG98_gp147 [Enterobacter phage PG7]		66
245	-	hypothetical protein	hypothetical protein CG98_gp294 [Enterobacter phage PG7]		74
246	-	hypothetical protein	hypothetical protein KAALPHA_159 [Klebsiella phage vB_KaeM_KaAlpha]	NADAR superfamily	149
247	-	Phage protein	hypothetical protein KAALPHA_160 [Klebsiella phage vB_KaeM_KaAlpha]		76
248	-	hypothetical protein	hypothetical protein [Enterobacter phage vB-EclM_KMB17]		73
249	-	hypothetical protein	hypothetical protein [Enterobacter phage vB-EclM_KMB20]		165
250	-	hypothetical protein	hypothetical protein CG98_gp289 [Enterobacter phage		124

251	-	hypothetical protein	hypothetical protein [Enterobacter phage vB-EclM_KMB20]		74
252	-	hypothetical protein	hypothetical protein CC31p142 [Enterobacter phage CC31]		119
253	-	hypothetical protein	hypothetical protein CG98_gp284 [Enterobacter phage PG7]		65
254	-	predicted membrane protein	predicted membrane protein [Enterobacter phage CC31]		59
255	-	Ip7 protein	Ip7 protein [Enterobacter phage CC31]		102
256	-	hypothetical protein	hypothetical protein CC31p147 [Enterobacter phage CC31]	57B superfamily	152
257	-	Phage tail fiber assembly protein	gp57A chaperone for tail fiber formation [Enterobacter phage CC31]	GP57 superfamily	84
258	-	deoxynucleoside monophosphate kinase	gp1 dNMP kinase [Enterobacter phage CC31]	NK superfamily	242
259	-	Phage tail completion protein	tail completion and sheath stabilizer protein [Enterobacter phage PG7]	3 superfamily	196
260	-	DNA end protector protein	DNA end protector protein [Enterobacter phage myPSH1140]	2 superfamily	276
261	-	head completion protein	head completion protein [Enterobacter phage PG7]	Tn7_Tnp_TnsA_N superfamily	149
262	+	baseplate wedge subunit	gp53 base plate wedge completion [Enterobacter phage CC31]	Phage_gp53 superfamily	191
263	+	Phage baseplate hub structural protein / Phage lysozyme R	baseplate hub subunit and tail lysozyme [Enterobacter phage myPSH1140]	5 superfamily	578

(E

264	+	hypothetical protein	hypothetical protein [Enterobacter phage vB-EclM_KMB20]	5.1 superfamily	167
265	+	hypothetical protein	hypothetical protein CC31p156 [Enterobacter phage CC31]	PAAR_like superfamily	98
266	+	baseplate wedge subunit	base plate wedge [Enterobacter phage PG7]	Baseplate_J superfamily	648
267	+	Phage baseplate wedge initiator (T4-like gp7)	baseplate wedge subunit [Enterobacter phage PG7]	7 superfamily	1032
268	+	Phage baseplate wedge subunit (T4-like gp8)	putative baseplate wedge subunit [Klebsiella phage vB_KaeM_KaAlpha]	Phage-Gp8 superfamily	336
269	+	Phage baseplate wedge tail fiber connector (T4-like gp9)	base plate wedge completion tail fiber socket [Enterobacter phage PG7]	T4_gp9_10 superfamily	302
270	+	baseplate wedge subunit and tail pin	putative baseplate wedge subunit and tail pin [Klebsiella phage vB_KaeM_KaAlpha]	10 superfamily	602
271	+	Phage baseplate wedge subunit (T4-like gp10)	Phage base plate wedge completion tail pin (T4-like gp11) [Cronobacter phage Pet-CM3-4]	GP11 superfamily	220
272	+	hypothetical protein	short tail fibers protein [Enterobacter phage PG7]	Collar superfamily	516
273	+	Phage fibritin neck whisker protein	Phage fibritin neck whisker protein [Cronobacter phage Pet-CM3-4]	Fibritin_C superfamily	472
274	+	Phage neck protein	T4-like phage head completion, neck hetero-dimeric protein (T4-like gp13) [Cronobacter phage Pet-CM3-4]	13 superfamily	312
275	+	Phage neck protein	gp14 neck protein [Enterobacter phage CC31]	T4_neck-protein superfamily	266
276	+	Proximal tail sheath	tail sheath stabilizer and completion protein	T4-gp15_tss superfamily	276

		stabilization protein	[Enterobacter phage fGh-Ecl01]		
277	+	Phage terminase, small subunit	small terminase protein [Enterobacter phage PG7]	DNA_Packaging superfamily	165
278	+	Phage terminase, large subunit	putative large terminase [Klebsiella phage vB_KaeM_KaAlpha]	17 superfamily	610
279	+	Phage tail sheath	gp18 tail sheath protein [Enterobacter phage CC31]	18 superfamily	660
280	+	tail fibers protein	tail protein [Enterobacter phage CC31]	19 superfamily	163
281	+	putative portal vertex protein	portal vertex of the head [Enterobacter phage vB-EclM_KMB17]	Peptidase_S80 superfamily	522
282	+	Phage prohead assembly (scaffolding) protein (T4-like gp67)	gp67 prohead core protein [Enterobacter phage CC31]	Gp67 superfamily	82
283	+	Phage capsid and scaffold	gp68 prohead core protein [Enterobacter phage CC31]	68 superfamily	141
284	+	Phage prohead assembly (scaffolding) protein	prohead core scaffolding protein and protease [Enterobacter phage CC31]	Peptidase_S77 superfamily	213
285	+	Phage prohead assembly (scaffolding) protein	prohead assembly (scaffolding) protein [Enterobacter phage vB-EclM_KMB17]	22 superfamily	271
286	+	major capsid protein	capsid protein [Enterobacter phage PG7]	Gp23 superfamily	520
287	+	capsid vertex protein	Phage capsid vertex [Cronobacter phage Pet-CM3-4]	Gp23 superfamily	427
repeat-2	+	repeat region			
288	-	hypothetical protein	hypothetical protein prasa_gp213 [Enterobacter phage prasa_myo]		32
